# MRI-derived diffusion parameters in the human optic nerve and its surrounding sheath during head-down tilt

**DOI:** 10.1038/s41526-017-0023-y

**Published:** 2017-06-21

**Authors:** Darius A. Gerlach, Karina Marshall-Goebel, Khader M. Hasan, Larry A. Kramer, Noam Alperin, Joern Rittweger

**Affiliations:** 1 Division of Space Physiology, Institute of Aerospace Medicine, German Aerospace Center (DLR), Cologne, Germany; 2 Neural Systems Group, Massachusetts General Hospital, Harvard Medical School, Charlestown, MA USA; 3 Department of Diagnostic and Interventional Imaging, University of Texas Health Science Center at Houston, McGovern Medical School, Houston, Texas USA; 4 Department of Radiology, University of Miami, Miami, FL USA; 5 Department of Pediatrics and Adolescent Medicine, University of Cologne, Cologne, Germany

## Abstract

More than half of astronauts present with significant neuro-ophthalmic findings during 6-month missions onboard the International Space Station. Although the underlying cause of this Microgravity Ocular Syndrome is currently unknown, alterations in cerebrospinal fluid dynamics within the optic nerve sheath may play a role. In the presented study, diffusion tensor imaging was used to assess changes in diffusivity of the optic nerve and its surrounding sheath during head-down tilt, a ground-based model of microgravity. Nine healthy male subjects (mean age ± SD: 25 ± 2.4 years; mean body mass index ± SD: 24.1 ± 2.4 kg/m^2^) underwent 5 head-down tilt conditions: −6°,−12°, −18°,−12° and 1% CO_2_, and −12° and lower body negative pressure. Mean diffusivity, fractional anisotropy, axial diffusivity, radial diffusivity were quantified in the left and right optic nerves and surrounding sheaths at supine baseline and after 4.5 h head-down tilt for each condition. In the optic nerve sheath, mean diffusivity was increased with all head-down tilt conditions by (Best Linear Unbiased Predictors) 0.147 (SE: 0.04) × 10^−3^ mm^2^/s (*P* < 0.001), axial diffusivity by 0.188 (SE: 0.064) × 10^−3^ mm^2^/s (*P* < 0.001), and radial diffusivity by 0.126 (SE: 0.04) × 10^−3^ mm^2^/s (*P* = 0.0019). Within the optic nerve itself, fractional anisotropy was increased by 0.133 (SE: 0.047) (*P* = 0.0051) and axial diffusivity increased by 0.135 (SE: 0.08) × 10^−3^ mm^2^/s (*P* = 0.014) during head-down tilt, whilst mean diffusivity and radial diffusivity were unaffected (*P* > 0.3). These findings could be due to increased perioptic cerebral spinal fluid hydrodynamics during head-down tilt, as well as increased cerebral spinal fluid volume and movement within the optic nerve sheath.

## Vision: Microgravity may alter optic nerve and surrounding sheath

Changes to the optic nerve and surrounding sheath during microgravity could explain why space flight is harmful to an astronaut’s vision. Darius Gerlach from the German Aerospace Center in Cologne and colleagues studied the tissue architecture of the optic nerve and its surrounding sheath in nine healthy men who experienced head-down tilt, a commonly used ground-based model of weightlessness. Using a neuroimaging technique called diffusion tensor imaging, the researchers documented fluid dynamic changes wrought by the microgravity-like conditions that could be due to alterations in the volume and movement of cerebrospinal fluid within and around the optic nerve. The findings may help explain why many astronauts experience poorer vision after long-duration space flights, although more work is needed to explore the effects of true microgravity on the visual system.

## Introduction

**Fig. 1:**
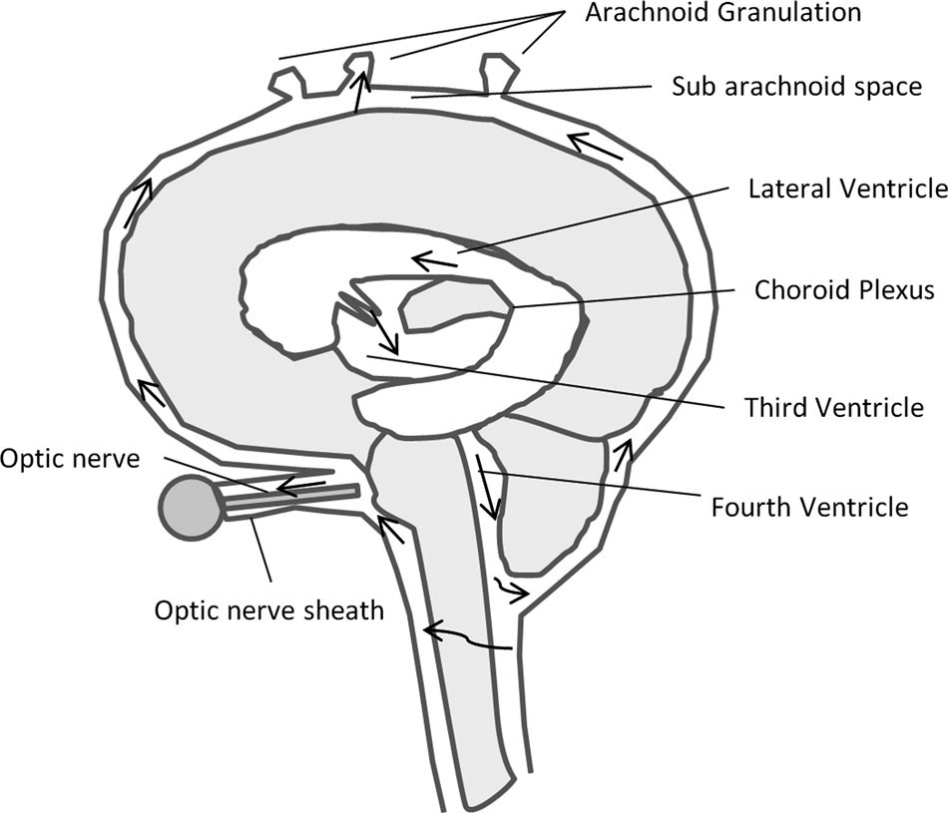
The current understanding of cerebrospinal fluid (CSF) dynamics is that the fluid is produced in choroid plexuses and moves from the third to the forth ventricle. The CSF then moves into the subarachnoid space, continuous along the optic nerve (ON) to the back of the eye. The Illustration is based on the original from the Intracranial Hypertension Research Foundation (www.IHRFoundation.org)

Changes in visual acuity during spaceflight have been found to affect ~ 29% astronauts during short-duration (~2 week) missions and ~ 60% astronauts during 6-month missions on-board the International Space Station (ISS). The hyperopic shift can be transient or result in permanent degradation of visual acuity or visual field loss.^1^ The underlying pathophysiological mechanism of this Microgravity Ocular Syndrome (MOS) is currently unknown,^2^ however, altered cerebral spinal fluid (CSF) dynamics in the subarachnoid space of the optic nerve (ON) sheath are hypothesized to play a role.^3^ CSF surrounds the brain, spinal cord and ON within the subarachnoid space and increased CSF pressure or altered fluid dynamics can result in damage to the ON and ON head, resulting in vision changes (Fig. 1).^1, 2, 4^

On a time-scale of minutes to hours, CSF pressure changes can result from alterations in its formation or resorption, the latter of which occurs primarily through the arachnoid granulations into the dural venous sinuses in a pressure-dependent way.^5, 6^ In addition, animal studies have found that a sizable amount of CSF could also be extruded via cranial nerves into the lymphatic system.^5, 7–10^ In mice, Furukawa et al. demonstrated lymphatic pathways in the dura matter of the ON by immunohistochemistry and electron microscopy.^11^ The link between intracranial, spinal, and ON sheath CSF is likely not a simple circuit, but rather a complex open system interacting with arterial and venous blood as well as lymphatics. Accordingly, CSF fluid dynamics are largely unknown, especially under microgravity conditions.^3^

Diffusion tensor imaging (DTI) is a valuable tool to assess the ON and its surrounding sheath at early pathological stages of various diseases before severe symptoms occur.^12–14^ By measuring the signal loss due to proton spin random translational motion, DTI can noninvasively examine microstructural changes and the integrity of white matter. Molecular diffusion captured in three dimensions provides information on both the orientation and magnitude including the fractional anisotropy (FA, the shape of diffusion), the mean diffusivity (MD, the magnitude of diffusion averaged over all directions), the axial diffusivity (AD, the magnitude of the main direction of diffusion) and the radial diffusivity (RD, the magnitude of diffusion perpendicular to the AD).

Several studies have found strong correlations between the severity of glaucoma and a reduction in FA, as well as an increase in MD in the ON.^12, 13, 15^ Additionally, AD and RD increased in the patient group.^12, 13^ Patients with optic neuritis have been found to have increased RD, MD, and decreased FA,^16^ with similar findings reported in patients with multiple sclerosis.^17^ In retinitis pigmentosa, higher MD, AD, and RD have been found compared to healthy controls, as well as lower FA.^14^ Moreover, CSF dynamics have been assessed with computer tomography cisternography in patients with glaucoma,^18^ as well as papilledema,^19^ and CSF compartmentation was reported as the cause for these alterations.

Thus, whilst DTI has provided valuable insight into disease-related alterations in the ON, it has not been used to study the physiological effects of sustained variations in intracranial pressure. Therefore, we aimed to determine the short-term effects of increased intracranial pressure in healthy subjects on ON diffusivity using the head-down tilt (HDT) model.^20^ Moreover, we aimed to differentiate between the ON and the perioptic CSF-filled ON sheath within the DTI acquisition. We hypothesized that HDT-induced cephalad fluid shifting would increase MD in the ON sheath due to altered CSF hydrodynamics, as potentially caused by pulsatile or cohesive CSF movement and thus causing signal loss from the phase shift.^21^ Finally, we were interested in whether lower-body negative pressure (LBNP), a technique known to reduce centrally available venous blood volume, and inspiratory carbon dioxide, a potent arterial vasodilator, in combination with HDT would have additional effects.

## Materials and methods

### Study design

This study was performed in compliance with the Declaration of Helsinki and was approved by the local ethics commission (Ärztekammer Nordrhein). Written and informed consent was obtained from all subjects prior to study inclusion. The study design has been previously reported.^22^ Briefly, nine healthy male subjects (mean age ± SD: 25 ± 2.4 years; mean height ± SD: 183 ± 6 cm; mean body mass index ± SD: 24.1 ± 2.4 kg/m^2^) participated in five experimental HDT conditions: −6°, −12°, −18°, −12° plus 1% CO^2^ atmosphere, and −12° plus LBNP. These five conditions were done in random order and on different experimental days. The LBNP was maintained at −20 mmHg during the entire 5 h HDT protocol. Magnetic Resonance Imaging (MRI) scans were performed at supine baseline after lying supine for at least 1 h and again after 4–5 h of intervention. Subjects were not allowed to sleep and remained in the HDT position during all experimental conditions. Custom-built MRI-compatible wedges in the three HDT positions, breathing masks and an LBNP chamber were used for the interventions. The HDT wedges ended at the neck of the subject, which coincided with the beginning of the head coil.

### Diffusion tensor imaging

The total duration of the MRI examination was ~1:05 h, with DTI performed at about ~30 min into the scan. MRI acquisitions were obtained on a 3 T scanner with a maximum gradient amplitude of 45 mT/m and maximum slew rate of 200 T/m/s (mMR Biograph—Positron Emission Tomography-Magnetic Resonance Imaging (PET-MRI) scanner based on the Verio system—Siemens, Erlangen, Germany) with an appendant 16 channel head and neck coil.

The orbital DTI parameters were: TR 5600 ms, TE 100 ms, FA 90°, Matrix 128 × 128, Filed of View 160 mm, 25 slices, NEX 2. The nominal resolution was 1.25 mm× 1.25 mm × 2.0 mm and the interpolated resolution was 0.625 mm × 0.625 mm × 2.0 mm, with an additional inter slice spacing of 2 mm. Thirty non-collinear diffusion directions with *b* value = 1000 s/mm^−2^ and one without diffusion weighted acquisition (*b* = 0 s/mm^−2^) were acquired. Parallel imaging with an acceleration factor of two was enabled (GRAPPA algorithm). A bandwidth of 1086 Hz/Px. with 1.03 ms echo spacing was applied. The DTI scan time was 6:04 min. A twice-focused single-shot spin-echo EPI was utilized to reduce geometric distortions.^23^ The phase encoding direction was A to P. To avoid foldover artefacts, a saturator of 55 mm was used in the posterior brain region. Scanner stability was assessed by spherical water phantom acquisition.^24^

**Fig. 2:**
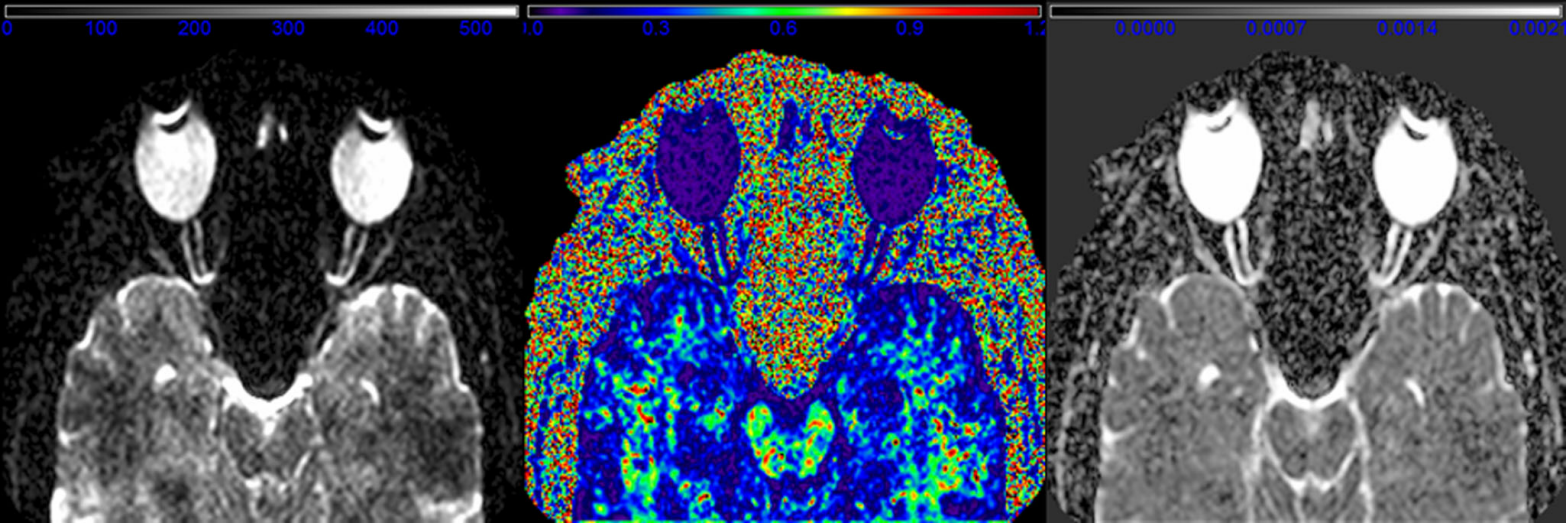
Sagittal oblique middle cut slice of orbital DTI of one subject. Left: non-diffusion weighted image with arbitrary grayscale, middle: fractional anisotropy (FA) map (colorbar ranges from 0 to 1.2), right: mean diffusivity (MD) map; diffusion intensity values are given in 10^−6^ mm^2^/s

High resolution anatomical T_2_ turbo spin echo (TSE) scans of left and right ON were used for the positioning of the orbital DTI. Oblique transversal sections through both ONs were planned in addition to a coronal magnetization-prepared rapid acquisition gradient-echo reconstruction. The positioning was further adjusted and controlled in the left and right sagittal TSE ON central sections. This procedure allowed for an exact adjustment of the DTI slices through both ONs (Fig. 2). In general, positioning was focused on the part of the ON closest to the eye to allow for optimal comparability in case of a curved shape or kinked ON. To avoid eye movement during DTI acquisition, which would result in artefacts, subjects were instructed to target their gaze on a defined spot throughout the scan. The spot was viewable for the subject through a mirror that was adjusted to allow for an anterior-posterior gaze alignment. The presented data represents a subset of data from a larger study.^22, 25^

**Fig. 3:**
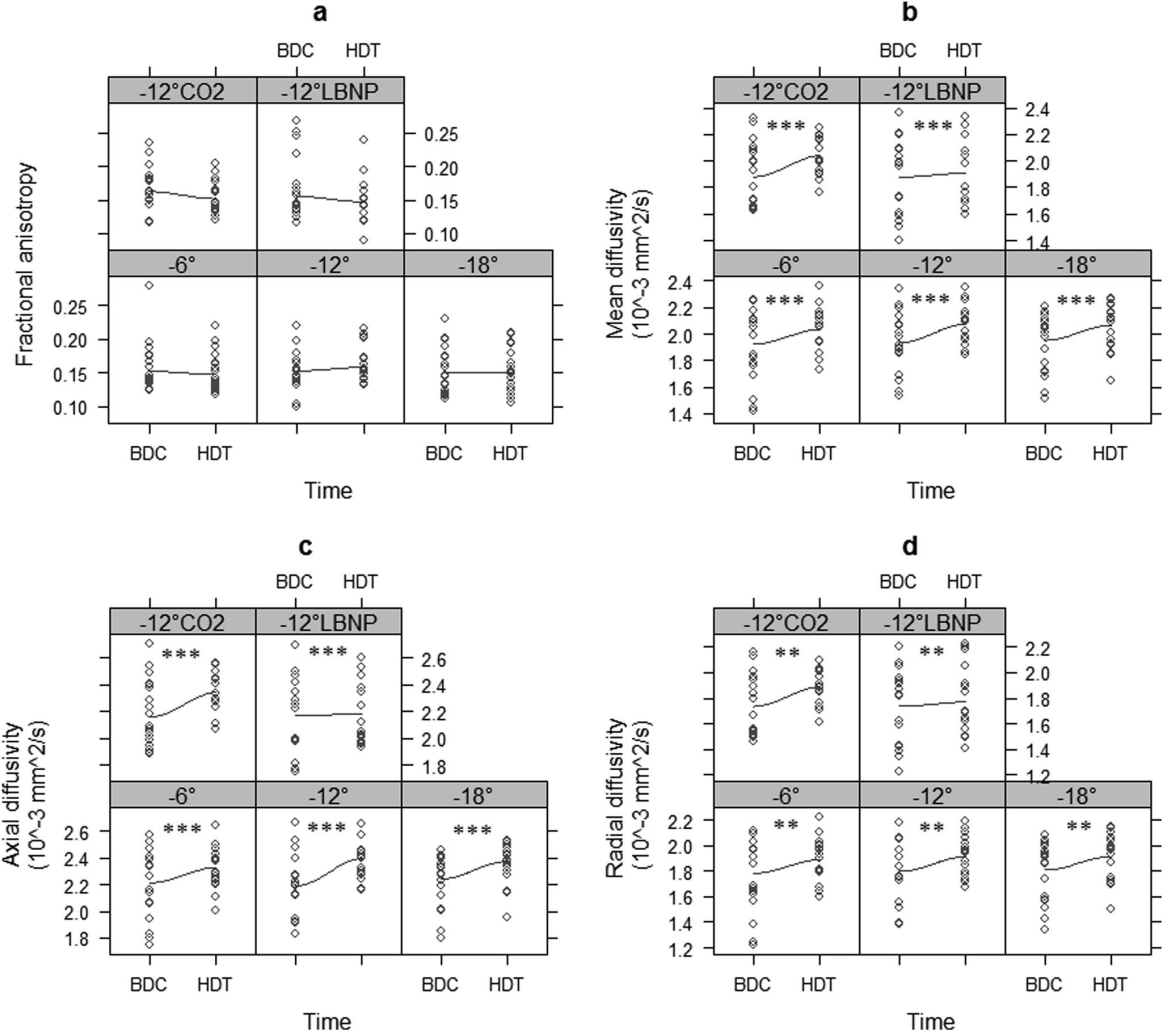
ON sheath diffusion parameters including FA **a**, MD **b**, axial diffusivity (AD) **c** and radial diffusivity (RD) **d** for all conditions (−6°, −12°, −18°, −12°, and CO2, −12° and LBNP) compared with the corresponding baseline value. *Asterisks* indicate significance level from linear mixed effects (LME) corresponding to time effect: *(*P* = 0.05), **(*P* < 0.01), ***(*P* < 0.001)

**Fig. 4:**
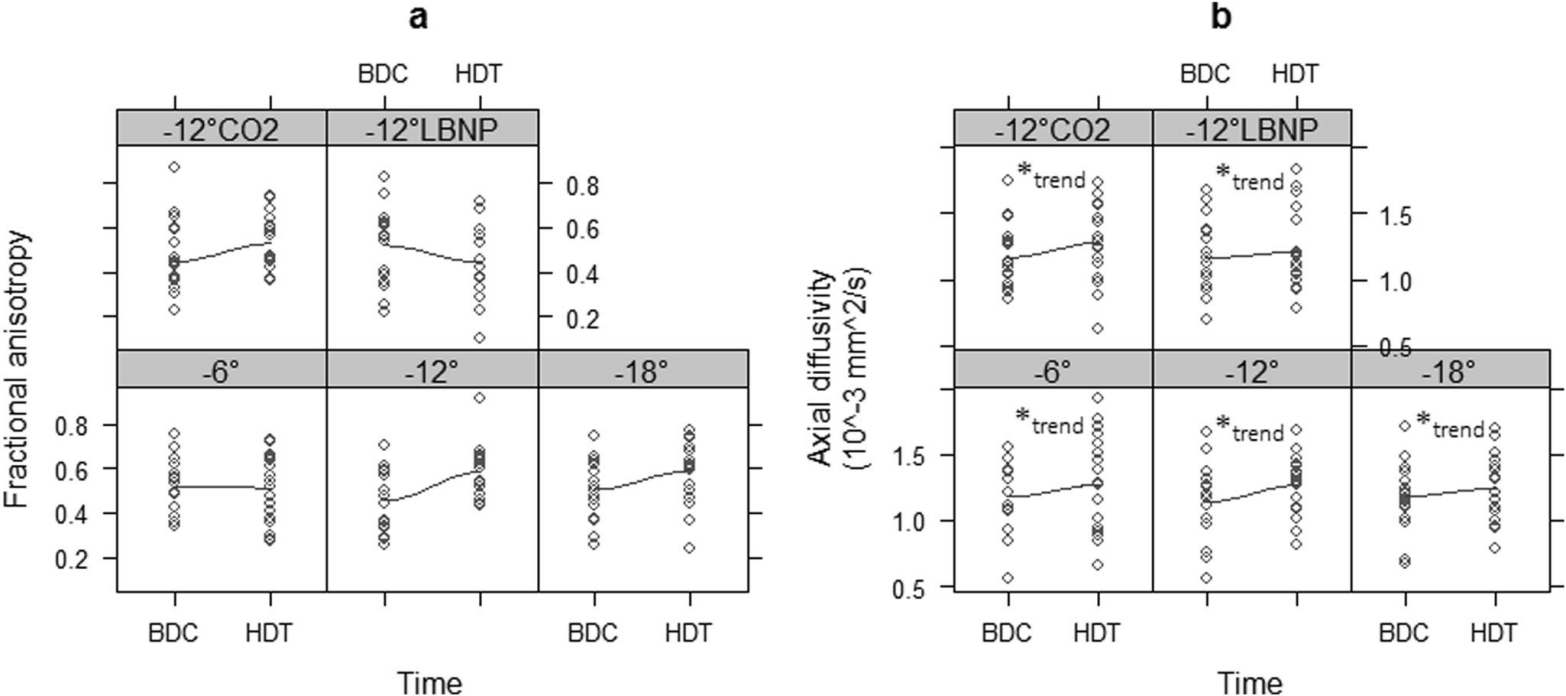
ON diffusion parameters including FA **a** and AD **b** for all conditions (−6°, −12°, −18°, −12° and CO_2_, −12° and LBNP) compared with the corresponding baseline value. *Asterisks* indicate significance level from LME corresponding to time effect: *(*P* = 0.05)

### Image analysis

DTI data was post-processed with FSL software library (http://www.fmrib.ox.ac.uk/fsl). Eddy current corrected diffusion weighted images were used for voxel-by-voxel based tensor calculation. The following parameter maps were calculated from the Tensor data: FA, MD (mean of eigenvalue *L*_1_, *L*_2_, and L_3_ (*L*_1_ + *L*_2_ + *L*_3_)/3), AD (equal to eigenvalue *L*_1_) and RD (mean of eigenvalue *L*_2_ and *L*_3_ (*L*_2_ + *L*_3_)/2)^26^:

$$\\ 	 FA  = \sqrt {\frac{3}{2}} \sqrt {\frac{{{{\left( {{L_1} - D} \right)}^2} + {{\left( {{L_2} - D} \right)}^2} + {{\left( {{L_3} - D} \right)}^2}}}{{L_1^2 + L_2^2 + {\it{L}}_3^2}}} \quad{\rm{with}}\,\\ \\ 	D = ({L_1} + {L_2} + {{\it{L}}_3})/3\quad\left( {26} \right)\\ \\ $$

The ON and its surrounding sheath were segmented manually with itk-SNAP (http://www.itksnap.org). 2D image planes of the middle ON section were utilized for segmentation. Depending on the shape of the ON, the segmented length was ~0.8 to 1.5 cm starting from the eye globe. The *B*_0_ non-diffusion weighted images were used as a reference for segmentation. FA, MD, AD, and RD values were calculated as mean values of the left and right ON and ON sheath region of interests.

### Statistical analysis

Statistical analysis was carried out with R statistical software version 3.2.0 (R Development Core Team, 2015) using ANOVA and linear mixed effects models to analyze the ON and ON sheath MD, FA, AD, and RD. Linear mixed effect models take care of inter-subject sources of variation and are thus suitable for repeated measurements. Time (baseline vs. 4.5 h HDT), side (left vs. right) and condition (−6°, −12°, −18°, −12°, and CO_2_, or −12° and LBNP) were used as fixed effects and subject as a random effect. Interactions of the main fixed effects were also included in the model, however, the three-way interaction term was removed in all instances during model simplification, as well as the term for side and interaction terms with it. Notably, the main effect of time then reflects effect of HDT, and the time × condition interaction reflects diversity of the different conditions over time. Significant overall ANOVA effects were inspected with a-priori defined treatment contrasts with −12° HDT as reference. Significance was set to *p* < 0.05. Means of the diffusion parameters (± standard deviation) for the ON sheath and ON MD, FA, AD, and RD are given for all conditions in Table 1 and Table 2, respectively. Best Linear Unbiased Predictors with standard error are shown in results text.

**Table 1:** Mean diffusivity (MD), fractional anisotropy (FA), axial diffusivity (AD), and radial diffusivity (RD) of CSF (cerebrospinal fluid) in the optic nerve (ON) sheath

	Baseline (0°)	−6°	−12°	−18°	−12° and CO_2_	−12° and LBNP
MD	1.910 ± 0.246	2.035^***^ ± 0.161	2.075^***^ ± 0.151	2.056^***^ ± 0.167	2.039^***^ ± 0.133	1.919^***^ ± 0.256
FA	0.162 ± 0.039	0.152 ± 0.029	0.163 ± 0.027	0.152 ± 0.033	0.154 ± 0.026	0.153 ± 0.044
AD	2.195 ± 0.240	2.326^***^ ± 0.150	2.398^***^ ± 0.148	2.353^***^ ± 0.148	2.341^***^ ± 0.137	2.193^***^ ± 0.236
RD	1.762 ± 0.252	1.889^**^ ± 0.171	1.914^**^ ± 0.156	1.907^**^ ± 0.182	1.887^**^ ± 0.136	1.781^**^ ± 0.271

MD, AD, RD are given in 10^−3^ mm^2^/s. FA is given as a ratio. Significant a-priori time contrast is indicated by asterisks: *(*P* = 0.05), **(*P* < 0.01), ***(*P* < 0.001)

**Table 2:** Mean diffusivity (MD), fractional anisotropy (FA), axial diffusivity (AD), and radial diffusivity (RD) of the optic nerve

	0° (Baseline)	−6°	−12°	−18°	−12° and CO_2_	−12° and LBNP
MD	0.775 ± 0.253	0.840 ± 0.346	0.757 ± 0.207	0.748 ± 0.254	0.797 ± 0.237	0.877 ± 0.343
FA	0.493 ± 0.146	0.511 ^*^ ± 0.153	0.592 ± 0.116	0.578 ± 0.143	0.533 ± 0.122	0.443 ^**^ ± 0.174
AD	1.165 ± 0.256	1.276 ± 0.366	1.261 ± 0.222	1.244 ± 0.248	1.264 ± 0.284	1.232 ± 0.313
RD	0.580 ± 0.264	0.621 ± 0.342	0.505 ± 0.213	0.500 ± 0.271	0.563 ± 0.225	0.699 ± 0.365

MD, AD, RD units are given in 10^−3^ mm^2^/s. FA is given as a ratio. Asterisks indicate significant time × condition interaction: *(*P* = 0.05), **(*P* < 0.01)

### Data availability

The datasets (MRI original files in DICOM format and post-procession steps in NIFTI format) generated during and analyzed during the current study are not publicly available due to containing personal details of subjects, but are available from the corresponding author on reasonable request.

## Results

With the exception of two scans (one −12° with LBNP, one baseline scan before −6° HDT), all data were acquired and analyzed as foreseen by the study protocol.

### ON sheath

Results for the ON sheath are given in Table 1. MD in the ON sheath revealed significant main effects of time (*P* < 0.001), suggesting that MD increased by 0.147 (SE: 0.04 × 10^−3^ mm^2^/s). There was also a significant effect of condition (*P* = 0.003), which however, did not reflect in any significant a-priori contrasts (all *P* > 0.17). Notably, there was no significant time × condition interaction (*P* = 0.37). Taken together, the results suggest that MD in the ON sheath was increased after exposure to HDT, with all HDT conditions having similar effects as the −12° condition. FA depicted a significant main effect of time (*P* = 0.036) and a trend for the time × condition interaction (*P* = 0.054). AD and RD, respectively, in the ON sheath revealed significant main effects of time (both *P* < 0.001), suggesting increases by 0.188 (SE 0.042) × 10^−3^ mm²/s for AD and 0.126 (SE 0.04) × 10^−3^ mm²/s for RD. Significant main effects of condition for AD and RD (*P* = 0.0016 and *P* = 0.0062, respectively) were inconclusive during inspection with a-priori contrasts (all *P* > 0.13), and there were no time × condition interaction effects found for AD and RD (*P* = 0.10 and *P* = 0.57, respectively. (Fig. 3).

### Optic Nerve

Results for ON diffusivity are given in Table 2. Here, a significant main effect of time was found for AD (*P* = 0.014), indicating that AD increased after exposure to HDT by 0.135 (SE 0.08) × 10^−3^ mm²/s. However, no effect of condition was found (*P* = 0.96), nor was there a time × condition interaction (*P* = 0.96), suggesting that time-related changes in the various HDT conditions were all comparable to the −12° condition. No significant main or interaction effects were found for MD or RD in the ON (all *P* > 0.3). For FA, there was no main effect of condition (*P* = 0.51), and a trend for time (*P* = 0.055). Moreover, FA depicted a time × condition interaction (*P* = 0.032, see Table 2). A-priori contrasts suggest that compared to −12° HDT, FA decreased by 0.138 (SE 0.067, *P* = 0.042) after −6° HDT compared to −12°HDT and by 0.207 (SE 0.068) after −12° plus LBNP. (Fig. 4).

## Discussion

In this study, we found DTI-derived ON sheath diffusivity to be enhanced during exposure to HDT, alluding to increased periorbital CSF hydrodynamics and increased CSF volume and movement during HDT. The greatest changes were observed in AD, and somewhat less in RD and MD. In the ON, conversely, effects of HDT exposure were observed for FA and AD, but not for RD or MD. Notably, the effects observed in this study were generally comparable between the −12° condition and the other conditions tested, except for FA in the ON sheath, which depicted some significant time × condition interactions. Thus, the initial hypotheses are generally confirmed by the results obtained.

### ON sheath diffusivity

Overall, we found increased MD, AD, and RD in the ON sheath, suggesting increased hydrodynamic CSF movement and CSF flow towards the perioptic space during HDT. This may be related to both pulsatile and cohesive CSF motion and resulting signal loss due to phase shift.^21^ The former interpretation is supported by the observation of increased CSF pulsatility measured by CINE phase-contrast MRI in the cerebral aqueduct during HDT.^27^ CSF pulsatility at the cerebral aqueduct may be a marker generalized increased intracranial CSF pulsatility, which may also be transmitted to the perioptic CSF space. Although DTI-derived parameters are not specific to micro-edema or increased pressure, HDT-induced headward fluid shifting and the resulting increase in ON sheath CSF volume and movement may contribute to ocular deformations, potentially also in the microgravity environment.

The rate of CSF resorption into the dural venous sinuses depends on the pressure difference between the CSF and the cerebral venous system.^28^ Previously reported phase-contrast MRI data of the present study demonstrated decreased arterial inflow and internal jugular venous outflow during HDT.^22^ Thus, a decrease in venous outflow and possible increase in cerebral venous pressure could disrupt CSF dynamics, increasing perioptic CSF volume and thereby indirectly affecting diffusion parameters found in the CSF space surrounding the ON. This interpretation is supported by the observation that MD, AD, and RD in the ON sheath are all increased to a comparable extent during HDT exposure. As mentioned, HDT-induced increases in ON sheath MD, AD, and RD were all comparable to effects of the −12° condition. This can potentially be explained by completed filling of the internal jugular veins, which is already reached with low tilt angles (i.e., −6° HDT;.^22^ The increase in MD and AD in the ON sheath may be indicative of CSF flow within the ON sheath due to HDT, possibly facilitated with perioptic CSF expansion. However, the observed increase in RD in the perioptic CSF space (Table 1) does not support the idea of a directed flow, but rather of a more undirected flow, potentially due to changes in CSF dynamics.

Furthermore, compartmentalization of CSF in the ON sheath space, which can be found in pathologies like glaucoma^18^ and papilledema,^19^ may also be a contributing factor to vision changes in space.

### ON diffusivity

Compared to baseline, MD and RD in the ON were unaffected by HDT, however, AD was found to increase, and increases in FA were dependent on the specific HDT conditions. Unlike in pathologies such as glaucoma, optic neuritis, multiple sclerosis, and retinitis pigmentosa where a decrease in FA is associated with integrity loss of white matter tracts due to cell damage, increasing FA and AD indicate the possibility of directed diffusion along the ON. Notably, results from this study are not indicative of a pathological reaction to short-term HDT, but rather a physiological reaction to HDT resulting in a higher fraction of water molecules moving along the ON axis. The present data suggest that HDT does not hinder, but rather fosters diffusivity in the perioptic space, and potentially also within the ON. However, the possibility of hampered perioptic hydrodynamics in microgravity is still unknown. HDT may not mimic all of the space-related physiological effects of cranial fluid regulation and the presented study investigated only short-term effects of HDT. Thus, to gain more insight into potential pathological processes in the ON and ON sheath secondary to microgravity exposure, DTI scans are needed pre-spaceflight and post-spaceflight missions on the ISS.

### Effects of LBNP/CO_2_

Although LBNP did not depict any substantial effects, apart from decreased FA compared to −12° within the ON, it needs to be considered that a relatively low LBNP (−20 mmHg) was utilized in this study due to the multiple hour exposure. LBNP has been shown to have sizable effects on intraocular pressure at −40 mmHg, whilst LBNP at −20 mmHg does not.^25^ Thus, further studies are warranted to determine the potential use of LBNP as a countermeasure to headward fluid shifting in relation to ocular changes in astronauts.

On the contrary, as an arterial vasodilator, increased ambient CO_2_ was hypothesized to further increase intracranial volume and thus pressure during HDT and elevate diffusivity further. However, exposure to increased ambient CO_2_ during HDT was not found to have any additional effects on diffusivity parameters of the ON or ON sheath.

### DTI values

The FA and MD baseline values of the ON found in this study are in line with previously reported values in healthy subjects.^15^ However, all diffusion values, especially AD, are slightly lower than previously reported values,^12, 14^ although there is great variation in the reported literature. Heterogeneity in reported diffusivity values in the literature may be the result of different age groups of healthy control subjects. In the presented study, young male subjects (25 ± 2.4 years) were enrolled and although not reported for healthy subjects, ON FA is known to decrease with age in patients with glaucoma.^15^ Furthermore, Wang et al. (2013) and Zhang et al. (2016) report diffusivity values for controls across a wide age range and mixed genders.^12, 14^ Interestingly, ocular changes in astronauts predominantly affects middle-aged men ^1, 4^ and thus, age could should be taken into consideration as a potential factor in MOS.

### Comparison with state of the art methods

Optical Coherence Tomography, cycloplegic refraction, fundus examinations, visual acuity, visual field thresholds are standard measures for astronauts ocular health pre and post flight according to the clinical practice guidelines of long-duration missions.^3, 29^ Choroidal folds, cotton wool spots, ON sheath distension, globe flattening, elevated CSF opening pressure or papilledema have been identified in 42% of 36 astronauts post-flight, with 19 examinations still pending.^1, 3, 4^

In the presented study, subjects did not suffer from significant visual impairment during or after the HDT. The time frame used in the presented study may be beneficial to understand perioperative visual loss due to ischemic optic neuropathy, seen in patients undergoing prolonged surgery requiring head-down positioning such as spinal,^30^ gynecological or robotic prostate surgery.^31^ Intraocular pressure assessed in patients with head-down (Trendelenburg) surgical positioning has been found to increase, however, retinal nerve fiber layer thickness measured with optical coherence tomography does not appear to be altered in patients with vision impairments after surgery.^32, 33^ Similarly, no clinically relevant findings were found during 14 day exposure to −6° HDT bed rest,^34^ whereas 70 day −6° HDT bed rest could provoke an increase in the superior, nasal, and inferior peripapillary retinal thickness.^35^

### Limitations and future considerations

There are several limitations inherent to the presented study that need to be taken into consideration when interpreting results. Notably, the study included a small number of subjects, a detriment that may affect statistical analysis, as well as interpretation. In addition, DTI of the ON has several limitations.^36, 37^ First, successful acquisition depends greatly on subject compliance as eye movement can result in blurred scans, resulting in an indefinable strong partial volume effect in DTI parameters. Furthermore, a long TA signal quality is limited by motion artifacts. This issue can be overcome by an eye triggered DTI examination, however, the problem persists with echo train lengths of 7–10 s. To prevent this, we chose to use high in-plane resolution with a lower slice thickness resolution. Prior to the DTI scans, we performed three high resolution anatomical scans of the orbits (transversal, coronal, and sagittal). This allowed the operator to adjust the DTI slices exactly through both ONs in the oblique transverse direction. This procedure, however, has two disadvantages: First, it is operator-dependent, and second, it is sensitive to subject movement which can cause partial volume artifacts. Therefore, we conclude that a scan with an isotropic resolution benefits from operator independence and insensitivity from subject movement between scans and no high resolution 3D cuts are needed for slice positioning. While a volumetric scan would be sufficient for slice positioning, sophisticated segmentation techniques would also be necessary. In addition, considering potential tortuosity of the ON, a DTI acquisition perpendicular (coronal direction) to the long axis is considered more robust to partial volume effects.^38^

Notably, the measurement technique may also be the cause of variation in reported DTI values compared to previously reported values in the literature. This may be due to motion artifacts, partial volume effects in the ON sheath or different acquisition approaches with fat and water suppression. The majority of related publications fail to describe how eye movement was avoided. Some studies have also used water and fat suppression techniques for ON scans to avoid signal acquisition from surrounding CSF spaces.^39, 40^ Even though the signal from the ON and CSF voxels is suppressed, partial volume effects cannot be avoided with a low resolution.

Another obstacle was the strong susceptibility for artifacts in the orbital region caused by tissue susceptibility differences between fatty tissue, bony structure, and the nasal cavities. A promising approach to avoid distortions by single shot echo planar imaging, especially at higher magnetic fields (i.e., 7T), is by utilizing spin echo-based sequences. These sequences are not affected by magnetic field inhomogeneities.^41, 42^ Advanced sequences such as diffusion-sensitized multishot rapid acquisition with relaxation enhancement could also be promising with higher magnetic fields resolution.^43^ The examination of deeper sections of the optic path (ON closer to the chiasm) would also benefit from such a non-distorted technique.

Finally, we would like to remind the reader that the implemented statistical approach tested for differences between any condition and the −12° condition. It is therefore possible that differences e.g. between −6° and −18° went unnoticed in the analyses, which would have provided further evidence to our understanding of HDT effects on perioptical hydrodynamics. In any case, tilt-angle dependent effects on ON DTI should be explored in more detail in future studies.

## Conclusions

Overall, the presented data constitute evidence to suggest facilitated diffusivity along the ON with HDT. This may indicate increased perioptic CSF hydrodynamics with increased CSF volume and/or movement within the ON sheath during HDT exposure. The sensitivity of DTI to detect altered physiology with HDT demonstrates its capability to be used to further investigate ocular changes in astronauts. This work can also be extended to the entire visual system including the optic chiasm, the optic tract, and radiations.^36^
